# Association Between Different Antiretroviral Therapy Regimens and Adipokine Secretion Profile in HIV-Infected Individuals

**DOI:** 10.3390/ijms27156878

**Published:** 2026-08-01

**Authors:** Beata Szymańska, Brygida Knysz, Agnieszka Piwowar

**Affiliations:** 1 Department of Toxicology, Faculty of Pharmacy, Wroclaw Medical University, Borowska 211, 50-556 Wroclaw, Poland; agnieszka.piwowar@umw.edu.pl; 2 Department of Infectious Diseases, Liver Diseases and Acquired Immune Deficiencies, Faculty of Medicine, Wroclaw Medical University, Koszarowa 5, 51-149 Wroclaw, Poland; brygida.knysz@umw.edu.pl

**Keywords:** HIV, combined antiretroviral therapy, adipokines

## Abstract

This study investigated the impact of human immunodeficiency virus (HIV) infection and combination antiretroviral therapy (cART) on adipokine concentrations, which are bioactive molecules secreted by adipose tissue and involved in the regulation of metabolism and inflammation. Alterations in adipokine levels may contribute to the metabolic disturbances observed in people living with HIV. The analyzed adipokine panel included resistin, visfatin, chemerin, angiopoietin-like protein 2 (ANGPTL2), lipocalin-2 (LCN2), Wnt family member 5A (Wnt5a), adiponectin, omentin, vaspin, secreted frizzled-related protein 5 (SFRP5), and apelin. Blood samples were collected from people living with HIV and HIV-negative control participants. Adipokine concentrations were measured using an enzyme-linked immunosorbent assay (ELISA). Patients were further stratified according to their cART regimen, including either protease inhibitor (PI)-based or integrase strand transfer inhibitor (INSTI)-based therapy. Plasma concentrations of ANGPTL2 and vaspin were significantly higher, whereas concentrations of visfatin, SFRP5, and adiponectin were significantly lower in HIV-infected patients compared with controls. Comparison of patients receiving INSTI- or PI-based regimens with the control group revealed significant differences in visfatin, SFRP5, and adiponectin concentrations. Notably, adiponectin concentrations were significantly lower in the INSTI-treated subgroup than in patients receiving PI-based therapy. These findings suggest that five of the examined adipokines may be associated with HIV infection and cART exposure, potentially contributing to the development of metabolic disturbances in this population. Further studies involving larger cohorts of individuals with HIV receiving long-term cART are required to better elucidate the relationship between adipokine alterations and the risk of treatment-related metabolic complications.

## 1. Introduction

Since its initial identification, human immunodeficiency virus (HIV) infection has remained a major global health concern affecting individuals across all age groups, despite substantial advances in prevention and treatment. The introduction and widespread implementation of combination antiretroviral therapy (cART) represented a major milestone in HIV management, extending the life expectancy of people living with HIV to levels approaching those of the general population [1]. Advances in cART have improved therapeutic efficacy and post-exposure prophylaxis strategies, resulting in a substantial reduction in new HIV infections, progression to acquired immunodeficiency syndrome (AIDS), and HIV-related mortality. However, despite effective viral suppression, people living with HIV continue to experience long-term consequences of infection, including metabolic complications and accelerated biological ageing [2,3]. HIV infection has been associated with epigenetic alterations, including changes in DNA methylation patterns, suggesting increased biological age compared with uninfected individuals. Although the mechanisms underlying this accelerated ageing remain incompletely understood, current evidence indicates that HIV-related immune dysfunction and chronic inflammation may contribute to this process, while the long-term effects of cART require further investigation [4,5].

Alterations in adipose tissue mass and distribution have been documented in individuals with HIV both prior to and following the initiation of cART. These changes include regional fat loss (lipoatrophy), abnormal fat accumulation (lipohypertrophy), and mixed forms of lipodystrophy. Such abnormalities may coexist with generalized obesity and reflect disturbances in energy homeostasis induced by HIV infection and antiretroviral treatment [6]. These findings suggest that adipose tissue contributes to HIV persistence and may play a role in maintaining chronic immune activation and inflammation in individuals receiving suppressive cART [7].

Adipose tissue is now recognized as a metabolically active endocrine organ that secretes a wide range of bioactive molecules. Among these molecules, adipokines produced by adipocytes exert diverse biological effects, including regulation of metabolism, inflammation, and immune responses. Depending on their specific functions, adipokines may exhibit either pro-inflammatory or anti-inflammatory properties, and disturbances in their balance may contribute to the development of metabolic disorders [8]. Therefore, alterations in adipokine secretion may represent an important mechanism linking HIV infection, cART exposure, and metabolic complications.

Integrase strand transfer inhibitor (INSTI)-based combination antiretroviral therapy (cART) is currently recommended as first-line treatment for most people living with HIV. However, accumulating evidence indicates that patients receiving INSTI-based regimens experience greater weight gain compared with those treated with protease inhibitor (PI)-based regimens [9]. Although the molecular mechanisms underlying INSTI-associated weight gain remain incompletely understood, they are thought to involve metabolic pathways promoting adipocyte hypertrophy, tissue hypoxia, and fibrosis [10].

The current literature provides limited information, with most studies focusing on only a few selected adipokines, such as leptin and adiponectin, in HIV-infected individuals receiving cART. Therefore, a broader characterization of adipokine profiles in this population remains warranted. In the present study, we investigated the plasma concentrations of both pro-inflammatory and anti-inflammatory adipokines in HIV-infected individuals receiving long-term antiretroviral therapy, taking into account the type of treatment regimen, and compared them with those of HIV-uninfected controls. The analyzed panel included the pro-inflammatory adipokines resistin, visfatin, chemerin, angiopoietin-like protein 2 (ANGPTL2), lipocalin 2 (LCN2), and Wnt family member 5A (Wnt5a), as well as the anti-inflammatory adipokines adiponectin, omentin, vaspin, secreted frizzled-related protein 5 (SFRP5), and apelin, which exhibits both pro- and anti-inflammatory properties. The primary objective of the study was to assess the associations between INSTI- and PI-based cART regimens and circulating adipokine concentrations in HIV-infected individuals. Additionally, we examined the associations between adipokine levels and selected immunological and biochemical parameters.

## 2. Results

### 2.1. Biochemical and Immunological Indicators in the Study Group

Table 1 presents the number of HIV-infected men depending on the values of biochemical and immunological indicators.

### 2.2. Assessment of Immunological Indicators in Study Group

The parameter results concerning the immune status of HIV-infected men are presented in Table 2.

### 2.3. Assessment of Demographic and Biochemical Indicators in Study and Control Group

All demographic and clinical data of the study and control groups are presented in Table 3.

Biochemical parameters, including age, fasting blood glucose (FBG), and total cholesterol (TC), did not differ significantly between the study and control groups (*p* > 0.05). However, high-density lipoprotein cholesterol (HDL-C) concentrations were significantly lower, whereas low-density lipoprotein cholesterol (LDL-C) concentrations were significantly higher in the study group compared with the control group.

### 2.4. A Panel of Adipokines in the Plasma in the Study and the Control Group with Statistical Analysis

The results and statistical analyses of adipokine levels in the study and control groups are presented in Table 4.

Analysis of adipokine concentrations revealed statistically significant differences in five parameters between HIV-infected men and individuals in the control group. The concentrations of angiopoietin-like protein 2 (ANGPTL2) and vaspin were significantly higher in the study group, with 1.3-fold and 1.4-fold increases, respectively, compared with controls. In contrast, the concentrations of visfatin, secreted frizzled-related protein 5 (SFRP5), and adiponectin were significantly lower in HIV-infected participants, showing 1.7-fold, 2.1-fold, and 1.4-fold decreases, respectively. The concentrations of Wnt5a, chemerin, omentin, and apelin showed an increasing trend in the study group; however, these differences did not reach statistical significance. Lipocalin-2 (LCN2) and resistin concentrations did not differ significantly between the two groups.

### 2.5. A Panel of Demographic, Immunological, and Biochemical Data of the Stud Group Based on the Type of cART

Table 5 presents the results of demographic, biochemical and immunological indicators obtained in subgroups of patients treated with two cART regimens.

No statistically significant differences were observed in biochemical or immunological parameters between patients receiving an NRTI + INSTI regimen and those receiving an NRTI + PI regimen.

Table 6 presents the concentrations of the analyzed adipokines according to the type of cART regimen, together with the results of the statistical analysis.

Comparative analysis of adipokine concentrations among patients receiving INSTI- or PI-based regimens and the control group revealed statistically significant differences for visfatin (K-W: H(2, N = 86) = 14.46, *p* < 0.001), SFRP5 (K-W: H(2, N = 86) = 7.19, *p* = 0.03), and adiponectin (K-W: H(2, N = 86) = 11.20, *p* = 0.004).

Visfatin concentrations were comparable between the INSTI- and PI-treated subgroups but were significantly lower in both treatment groups compared with the control group. Significant differences compared with the control group were observed only in the INSTI-treated subgroup. Adiponectin concentrations were significantly lower in the INSTI-treated subgroup compared with both the PI-treated subgroup and the control group.

No statistically significant differences were observed between the two treatment subgroups for the remaining adipokines.

### 2.6. Adipokine Correlations

Table 7 presents the results of the correlation analysis between adipokine concentrations and biochemical and immunological parameters in the study group, performed using Spearman’s rank correlation test.

Among the analyzed parameters, five adipokines—Wnt5a, ANGPTL2, SFRP5, omentin, and apelin—showed positive correlations with each other. Additional associations were identified between specific adipokines and clinical or biochemical parameters. Lipocalin-2 (LCN2) was positively correlated with HIV RNA levels, total cholesterol (TC), and low-density lipoprotein cholesterol (LDL) concentrations. Vaspin demonstrated positive correlations with body weight and body mass index (BMI), whereas Wnt5a and SFRP5 were negatively correlated with age.

### 2.7. Correlations in the Study Subgroup Depending on the Type of cART Regimen

Table 8 presents the correlations between adipokine concentrations and biochemical and immunological parameters, as well as age, body weight, and body mass index (BMI), in subgroups of patients receiving either INSTI- or PI-based cART regimens.

Correlations observed exclusively in subgroups of patients receiving different cART regimens, but not in the overall study group, are highlighted in grey in Table 7. Subgroup analysis revealed additional correlations in the INSTI-treated subgroup involving LCN2, visfatin, and chemerin, and in the PI-treated subgroup involving resistin, adiponectin, chemerin, and vaspin.

In the INSTI-treated subgroup, LCN2 concentrations were positively correlated with CD4^+^ T-cell counts, whereas SFRP5 concentrations were negatively correlated with CD8^+^ T-cell counts. In the PI-treated subgroup, Wnt5a concentrations were positively correlated with CD8^+^ T-cell counts and negatively correlated with the CD4^+^/CD8^+^ ratio, while ANGPTL2 concentrations were negatively correlated with CD4^+^ T-cell counts.

## 3. Discussion

HIV infection and combination antiretroviral therapy (cART) are associated with persistent alterations in adipose tissue distribution, metabolism, and chronic low-grade inflammation. Adipose tissue functions not only as an energy storage organ but also as an active endocrine and immunological compartment, producing numerous adipokines involved in metabolic regulation and immune responses. HIV infection, together with long-term exposure to antiretroviral drugs, has been shown to alter adipocyte biology and contribute to adipose tissue dysfunction, which may promote metabolic complications despite effective viral suppression [10,11,12].

In the present study, we evaluated circulating concentrations of pro- and anti-inflammatory adipokines in HIV-infected men receiving long-term cART and investigated their associations with biochemical, immunological, and anthropometric parameters. Significant differences were observed for five adipokines compared with healthy controls. Plasma concentrations of ANGPTL2 and vaspin were significantly higher, whereas adiponectin, visfatin, and SFRP5 were significantly lower in HIV-infected participants. Comparison of treatment regimens revealed relatively small differences between patients receiving InSTI- and PI-based cART, with significantly lower adiponectin and visfatin concentrations observed in the InSTI-treated subgroup. Additionally, visftin concentrations were significantly lower in the PI-treated subgroup compared to the control group. Concentrations of other adipokines did not differ between treatment regimens. 

Among the adipokines evaluated, adiponectin exhibited one of the most pronounced alterations. Plasma adiponectin concentrations were significantly lower in HIV-infected patients than in controls and were particularly reduced in individuals receiving INSTI-based therapy. Adiponectin is an anti-inflammatory adipokine involved in glucose and lipid metabolism, and decreased circulating concentrations have been associated with insulin resistance, dyslipidaemia, and increased cardiovascular risk [13].

Our findings are consistent with previous reports demonstrating reduced adiponectin concentrations in people living with HIV receiving cART, particularly among patients with lipodystrophy and metabolic abnormalities [14,15]. Klos et al. [14] observed lower adiponectin concentrations in HIV-infected individuals receiving antiretroviral therapy. Tomono et al. [15] demonstrated that lower adiponectin concentrations were associated with an adverse lipid profile, supporting the hypothesis that adiponectin dysregulation may contribute to the increased cardiometabolic risk observed in people living with HIV.

In the present study, the lowest adiponectin concentrations were observed in the INSTI-treated subgroup. Although the cross-sectional design precludes causal inference, these findings suggest that INSTI-based therapy may be associated with more pronounced adiponectin dysregulation and support the need for further longitudinal studies to clarify the relationship between antiretroviral therapy, adiponectin, and metabolic complications.

Plasma visfatin levels were significantly lower in our HIV cohort than in the control group, both in patients receiving INSTI- and PI-based therapy. This finding differs from that of Schindler et al. [16], who reported increased visfatin concentrations after one year of cART, despite unchanged glucose metabolism and adipose tissue mass, with visfatin levels positively correlating with the HOMA-IR index.

The discrepancy between the studies may be explained by differences in treatment duration, antiretroviral regimens, and patient characteristics. While Schindler et al. [16] evaluated patients after one year of cART, our cohort consisted of individuals receiving long-term contemporary cART, predominantly INSTI- or PI-based regimens. These findings suggest that prolonged antiretroviral therapy may differentially influence visfatin secretion, although the clinical significance of reduced visfatin concentrations requires confirmation in larger prospective studies.

Plasma ANGPTL2 concentrations were significantly higher in HIV-infected patients than in healthy controls, whereas no differences were observed between the INSTI- and PI-treated subgroups. ANGPTL2 concentrations correlated positively with apelin, omentin, SFRP5, and Wnt5a, while a negative correlation with CD4^+^ T-cell count was observed in the PI-treated subgroup.

ANGPTL2 is a pro-inflammatory adipokine involved in chronic low-grade inflammation, endothelial dysfunction, tissue remodeling, and the development of insulin resistance and other metabolic disorders. Increased circulating ANGPTL2 concentrations have also been associated with cardiovascular and renal complications, suggesting that this adipokine may serve as a marker of persistent metabolic and inflammatory disturbances.

Few studies have evaluated ANGPTL2 in people living with HIV. Pinzone et al. [17] reported an inverse association between circulating ANGPTL2 concentrations and renal function in patients receiving cART for at least one year, indicating its potential value as a biomarker of HIV-related complications. Although direct comparison is limited by differences in study populations, our findings similarly suggest that elevated ANGPTL2 concentrations persist despite long-term antiretroviral therapy. Moreover, the negative association with CD4^+^ T-cell count supports a potential link between ANGPTL2 and residual immune dysregulation in treated HIV infection.

Data regarding vaspin in people living with HIV remain limited. In the present study, plasma vaspin concentrations were significantly higher in HIV-infected patients than in controls, irrespective of the antiretroviral regimen. 

Vaspin is an anti-inflammatory, insulin-sensitizing adipokine involved in the regulation of glucose metabolism and adipose tissue function. Experimental studies suggest that vaspin suppresses the production of pro-inflammatory adipokines while promoting adiponectin secretion [18,19]. Increased circulating vaspin concentrations have also been described in obesity and type 2 diabetes and are associated with adverse metabolic parameters, including increased BMI, body fat, triglyceride, and insulin concentrations [20]. Therefore, the elevated vaspin concentrations observed in our cohort may reflect a compensatory response to persistent metabolic stress and chronic inflammation despite long-term cART rather than a treatment-specific effect.

To our knowledge, this is the first study to evaluate circulating SFRP5 and Wnt5a concentrations in people living with HIV receiving contemporary antiretroviral therapy. We observed significantly lower SFRP5 concentrations in HIV-infected patients than in healthy controls, whereas Wnt5a concentrations tended to be higher, particularly in the INSTI-treated subgroup. 

SFRP5 and Wnt5a are key regulators of the non-canonical Wnt signaling pathway. Wnt5a promotes inflammation and insulin resistance through activation of the JNK pathway, whereas SFRP5 antagonizes Wnt5a signaling and exerts anti-inflammatory effects [21,22]. The reduced SFRP5 concentrations observed in our study may therefore indicate impaired anti-inflammatory regulation despite long-term cART. In addition, experimental evidence suggests that Wnt signaling contributes to HIV latency by regulating viral transcription in CD4^+^ T cells [23]. Although the relationship between circulating SFRP5/Wnt5a and HIV persistence remains to be established, our findings suggest that dysregulation of this signaling axis may represent a novel feature of chronic treated HIV infection, linking adipose tissue inflammation with persistent immune activation.

Circulating chemerin, omentin, apelin, resistin and LCN2 concentrations did not differ significantly between HIV-infected patients and healthy controls or between INSTI- and PI-treated subgroups, suggesting that long-term cART does not substantially affect their circulating levels. Chemerin is involved in immune cell recruitment and metabolic regulation [24,25], whereas omentin contributes to insulin sensitivity and glucose metabolism, with reduced concentrations previously associated with obesity, insulin resistance, and metabolic disorders [26,27,28]. In people living with HIV, lower omentin concentrations have been reported in patients with lipodystrophy; however, our findings did not demonstrate significant alterations according to HIV status or treatment regimen [29].

Although apelin and LCN2 concentrations were not significantly different between groups, their correlations with other adipokines and clinical parameters may indicate involvement in adipose tissue-related metabolic and inflammatory pathways during treated HIV infection. Apelin, which regulates energy metabolism and insulin sensitivity and has been implicated in HIV pathogenesis through its receptor function [30,31], showed associations with several adipokines, including SFRP5, Wnt5a, ANGPTL2, and omentin. 

LCN2, a pro-inflammatory adipokine associated with metabolic disorders [32], has previously been reported to be elevated in people living with HIV despite cART [33]. In our cohort, LCN2 was not altered between groups but correlated with selected clinical parameters, suggesting that its role in long-term treated HIV infection requires further investigation.

The results of correlation analysis indicate the coexistence of changes in the concentrations of the studied adipokines and their associations with selected clinical and biochemical parameters. The observed relationships suggest that individual adipokines may reflect different aspects of metabolic disorders and disease activity in people living with HIV.

In patients treated with INSTIs, relationships were observed that may indicate a more favorable immune profile. The positive correlation of LCN2 concentration with CD4^+^ T cell count may suggest a link between this marker and better immune reconstitution, while the negative correlation of SFRP5 with CD8^+^ lymphocyte count indicates a potential role of this protein in regulating the immune response. In patients treated with PIs, the relationships were more closely related to markers of immune activation. The positive correlation of Wnt5a with CD8^+^ lymphocyte count and the negative correlation with the CD4^+^/CD8^+^ ratio may reflect persistent immune activation or less favorable immune reconstitution. Additionally, the negative correlation of ANGPTL2 with CD4^+^ lymphocyte count may indicate that higher levels of this protein are associated with poorer immune status.

### Strengths and Limitations

The present study has several strengths. It represents one of the most comprehensive analyses of circulating adipokines in HIV-infected men receiving long-term cART and, to our knowledge, is the first to investigate the SFRP5/Wnt5a axis in this population. Simultaneous assessment of multiple pro- and anti-inflammatory adipokines enabled a broader evaluation of adipose tissue dysfunction associated with chronic treated HIV infection.

Several limitations should also be acknowledged. First, the cross-sectional design precludes conclusions regarding causality. Second, metabolic characterization was limited, as measurements of fasting insulin, HOMA-IR, waist circumference, total body fat, and regional fat distribution were not available. Inclusion of these parameters would have provided a more comprehensive assessment of insulin resistance and body composition. Finally, despite an increasing number of studies investigating adipokines in HIV infection, evidence remains limited for several of the proteins evaluated, particularly SFRP5 and Wnt5a, making direct comparison with previous reports difficult.

Although the sample size was relatively modest (n = 53), assembling such a homogeneous group was challenging, particularly because all participants had received continuous cART for at least five years and had no significant comorbidities. We believe that this homogeneity strengthens the internal validity of the study.

The relatively small sample size and the number of analyzed biomarkers limited the feasibility of performing multivariable analyses. Future studies including larger cohorts and longer follow-up periods are needed to determine independent predictors of adipokine alterations and their relationship with metabolic and immunological outcomes in people living with HIV.

## 4. Material and Methods

### 4.1. Study Group Characteristics

The study group consisted of 53 men infected with HIV-1, with a mean age of 39.5 years (range: 26–59 years). All participants were receiving cART and were under medical care at the Center for Addiction Prevention and Therapy in Wroclaw and the Clinic of Infectious Diseases, Liver Diseases, and Acquired Immune Deficiencies of the Medical University of Wroclaw.

Inclusion criteria were: confirmed HIV infection and continuous cART use for at least five years. Exclusion criteria included co-infection with hepatitis B or C viruses, the presence of malignancies, other chronic diseases (including diabetes, hypertension, or urinary tract diseases), and the use of additional treatments.

Data regarding immune status, including CD4^+^ and CD8^+^ T lymphocyte counts, CD4^+^/CD8^+^ ratio, HIV viral load, and biochemical such parameters as: fasting blood glucose (FBG), total cholesterol (TC), low-density lipoprotein (LDL), high-density lipoprotein (HDL), and triglycerides (TG), as well as body weight and body mass index (BMI), were obtained from patient medical records.

Following HIV diagnosis, all study participants received one of two cART regimens for at least five years. The first included a protease inhibitor (PI) regimen: ritonavir- or cobicistat-boosted darunavir in combination with nucleoside reverse transcriptase inhibitors (NRTIs), emtricitabine and tenofovir alafenamide. The second regimen included an integrase strand transfer inhibitor (INSTI): dolutegravir combined with the same NRTIs.

The control group comprised 33 male volunteers, primarily from Wroclaw and the surrounding area, with a mean age of 41 years (range: 33–47 years). Controls were free of chronic or inflammatory diseases, including kidney disease, cardiovascular disease, diabetes, and hepatitis B or C, and were not receiving any chronic medication. Biochemical parameters and BMI were measured in the control group; immune status parameters were not determined.

The study was conducted in accordance with the Declaration of Helsinki, and the protocol was approved by the Bioethical Committee of the Medical University of Wroclaw (Nr KB 133/2026).

### 4.2. Material for Research

The research material was plasma obtained from whole blood, which was collected from fasting subjects into test tubes with an anticoagulant (tubes with EDTA, Kremsmunster, Austria). The tubes were centrifuged in an MPW-350 laboratory centrifuge (MPW Instruments, Warsaw, Poland) at 1500× *g* for 10 min to separate the plasma. The plasma was placed in microtubes (Eppendorf AG, Hamburg, Germany) and stored at −80 °C until the start of the study.

### 4.3. Test Methods

The concentrations of the tested parameters were measured using enzyme-linked immunosorbent (ELISA) methods. All tests were performed in accordance with the manufacturers’ instructions. The following tests included the Wnt5a (Cat. No SL3856Hu, SunLong Biotech Co., LTD., Hangzhou, China; Precision: Intra-Assay: CV < 10%; Inter-Assay: CV < 12%; Assay range: 30 pg/mL−2000 pg/mL; Sensitivity 6 pg/mL), ANGPTL2 (Cat. No SL2560Hu, SunLong Biotech Co., Ltd. Zhejang, China; Precision: Intra-Assay: CV < 10%; Inter-Assay: CV < 12%; Assay range: 56 pg/mL−4000 pg/mL; Sensitivity 12 pg/mL), Visfatin (Cat. No CSB-E08940h, Cusabio, Wuhan, China; Precision: Intra-Assay: CV < 8%; Inter-Assay: CV < 10%; Assay range: 0.625 ng/mL−40 ng/mL; Sensitivity 0.156 ng/mL), LCN2 (Cat. No EL3510-1, Assaypro LLC, St. Charles, MO, USA, Precision: Intra-Assay: CV < 10%; Inter-Assay: CV < 10%; Assay range: 0.063 ng/mL–2 ng/mL; Sensitivity 0.01 ng/mL), Resistin (Cat. No E50, Mediagnost, Reutlingen, Germany; Precision: Intra-Assay: CV < 10%; Inter-Assay: CV < 10%; Assay range: 0.02 ng/mL–1 ng/mL; Sensitivity 0.012 ng/mL), Chemerin (Cat. NoE102, Mediagnost, Reutlingen, Germany; Precision: Intra-Assay: CV < 2.17%; Inter-Assay: CV < 5.16%; Assay range: 25 pg/mL–600 pg/mL; Sensitivity 0.005 pg/mL), SFRP5 (Cat. No SL2192Hu, SunLong Biotech Co., Hangzhou, China; Precision: Intra-Assay: CV < 10%; Inter-Assay: CV < 12%; Assay range: 0.3 ng/mL–20 ng/mL; Sensitivity 0.05 pg/mL), Adiponectin (Cat. No E09, Mediagnost, Reutlingen, Germany; Precision: Intra-Assay: CV < 5%; Inter-Assay: CV < 7.5%; Assay range: 0.094 ng/mL–0.59 ng/mL; Sensitivity 0.27 ng/mL), Vaspin (Cat. No E106, Mediagnost (Reutlingen, Germany; Precision: Intra-Assay: CV < 1.4%; Inter-Assay: CV < 4.7%; Assay range: 0.027 ng/mL–1 ng/mL; Sensitivity 0.4 pg/mL), Omentin (Cat. No SL1293Hu, SunLong Biotech Co., LTD., Hangzhou, China; Precision: Intra-Assay: CV < 10%; Inter-Assay: CV < 12%; Assay range: 3 pg/mL−200 pg/mL; Sensitivity 0.6 pg/mL), Apelin (Cat. No SL0277Hu, SunLong Biotech Co., LTD., Hangzhou, China; Precision: Intra-Assay: CV < 10%; Inter-Assay: CV < 12%; Assay range: 35 pg/mL−1600 pg/mL; Sensitivity 16 pg/mL).

Parameter concentration measurements were performed in duplicate using plasma samples that had been thawed only once. The concentration values for all analyzed samples were within the assay measurement range.

Adipokine concentrations were measured using a sandwich enzyme-linked immunosorbent assay (ELISA). Each adipokine was immobilized with a human-specific monoclonal antibody and detected using a human-specific polyclonal antibody conjugated to horseradish peroxidase. The enzymatic reaction was developed with a substrate solution (3,3′,5,5′-tetramethylbenzidine [TMB] or chromogen A and B), then stopped, and absorbance was measured at 450 nm using an ELISA reader. Concentrations were calculated using standard curves, either by linear regression or point-to-point plotting of standard concentrations against absorbance. Final adipokine concentrations were adjusted according to the applied dilution factors.

Absolute counts of CD4^+^ and CD8^+^ T lymphocytes were determined from whole blood collected in EDTA tubes (Sarstedt, Poland). CYTO-STAT tri-CHROME CD8-FITC/CD4-RD1 reagents (Beckman Coulter, Brea, CA, USA) containing mouse monoclonal antibodies were used to simultaneously identify and quantify total CD4^+^ and CD8^+^ T cells. Samples were analyzed on a Navios EX flow cytometer (Beckman Coulter, Brea, CA, USA).

HIV-1 RNA viral loads were quantified using the Cobas^®^ HIV-1 real-time PCR assay (Roche Diagnostics GmbH, Mannheim, Germany) on the Cobas^®^ 4800 system. The lower limit of detection for the assay was 40 HIV RNA copies/mL.

### 4.4. Statistical Analysis

All statistical analyses were performed using Statistica version 13.3 (TIBCO Software Inc., Palo Alto, CA, USA, 2017). Data distribution was assessed using the Shapiro–Wilk test. Given the nonparametric nature of the data, comparisons between two groups were conducted using the Mann–Whitney U test, while comparisons among multiple subgroups and the control group were performed using the Kruskal–Wallis test. Correlations between parameters were analyzed using Spearman’s rank correlation coefficient. A *p*-value of less than 0.05 was considered statistically significant. Spearman correlation coefficients (R) were interpreted as follows:

- 0.3 ≤ R < 0.5: low correlation, clear dependence;
- 0.5 ≤ R < 0.7: high correlation, significant dependence;
- 0.7 ≤ R < 0.9: very high correlation, very strong dependence;
- 0.9 ≤ R < 1.0: full correlation, near-complete dependence.

Given the exploratory nature of the study and the relatively limited sample size, no formal adjustment for multiple comparisons was applied. The results are intended to identify potential associations that warrant further investigation in larger, independent studies.

## 5. Conclusions

In conclusion, HIV-infected men receiving long-term cART exhibit persistent alterations in circulating adipokine profiles despite effective treatment. Reduced concentrations of adiponectin, visfatin, and SFRP5 together with increased ANGPTL2 and vaspin suggest sustained adipose tissue dysfunction and low-grade inflammation. Particularly noteworthy is the dysregulation of the SFRP5/Wnt5a axis, which may represent a novel pathway linking adipose tissue inflammation, metabolic disturbances, and immune regulation in treated HIV infection. Larger prospective studies incorporating detailed metabolic phenotyping are needed to determine whether these adipokines can serve as biomarkers of treatment-related metabolic complications or therapeutic targets in people living with HIV.

## Figures and Tables

**Table 1:** Biochemical and immunological indicators in the study group.

Biochemical and Immunological Indicators	Study Group	
	Value	n (%)
FBG [mg/dL]	≥99	13 (25)
TC [mg/dL]	≥190	18 (34)
LDL [mg/dL]	≥115	22 (42)
HDL [mg/dL]	≥40	16 (30)
TG [mg/dL]	≥150	12 (23)
BMI [kg/m^2^]	≥25	21 (40)
T CD4+ lymphocytes [cells/μL]	≥500	47 (89)
T CD8+ lymphocytes [cells/μL]	≥500	31 (58)
CD4+/CD8+ ratio	≥1	18 (34)
HIV RNA [copies/mL]	<50	

Abbreviations: BMI—body mass index, calculated as weight in kilograms divided by height in meters squared; FBG—fasting blood glucose concentration; HDL—high-density lipoprotein cholesterol; IQR—interquartile range, representing the range between the 25th and 75th percentiles of the data distribution; LDL—low-density lipoprotein cholesterol; n—number of participants included in the analysis; TC—total cholesterol concentration; TG—triglyceride concentration.; CD4^+^ T lymphocytes—cluster of differentiation 4-positive T helper lymphocytes, key immune cells involved in immune regulation and antiviral responses; CD8^+^ T lymphocytes—cluster of differentiation 8-positive cytotoxic T lymphocytes, responsible for the elimination of infected cells; CD4^+^/CD8^+^ ratio—ratio of CD4^+^ helper T lymphocyte count to CD8^+^ cytotoxic T lymphocyte count, used as an indicator of immune system balance; cells/μL—number of cells per microliter of blood.

**Table 2:** Results of immunological indicators in a group of HIV-infected men.

Immunological Indicators	Mean ± SD Median (IQR)
CD4+ T lymphocytes [cells/µL]	658.88 ± 251.81 657 (500.00–754.00)
CD8+ T lymphocytes [cells/µL]	707.93 ± 248.91 674.500 (530.00–823.00)
CD4+/CD8+ ratio	1.03 ± 0.5 0.92 (074.00–1.32)

Abbreviations: IQR—interquartile range representing the range between the 25th and 75th percentiles of the data distribution; SD—standard deviation; CD4^+^ T lymphocytes—cluster of differentiation 4-positive T helper lymphocytes, key immune cells involved in immune regulation and antiviral responses; CD8^+^ T lymphocytes—cluster of differentiation 8-positive cytotoxic T lymphocytes, responsible for the elimination of infected cells; CD4^+^/CD8^+^ ratio—ratio of CD4^+^ helper T lymphocyte count to CD8^+^ cytotoxic T lymphocyte count, used as an indicator of immune system balance; cells/μL—number of cells per microliter of blood.

**Table 3:** Demographic and biochemical indicators of study and control group with statistical analysis.

Indicators	Study Group (n = 53)	Control Group (n = 33)	*p* *
Mean ± SD Median (IQR)			
Age [years]	39.47 ± 7.71 38.00 (34.00–46.00)	40.82 ± 4.04 40.50 (38.00–43.50)	0.156
FBG [mg/dL]	94.54 ± 15.3 91.00(86.00–103.00)	93.35 ± 10.33 94.10 (88.00–99.10)	0.688
TC [mg/dL]	189.95 ± 37.63 183.00 (167.00–216.00)	178.5 ± 21.10 179.00 (164.00–195.00)	0.208
LDL [mg/dL]	127.87 ± 42.75 121.00 (107.00–134.00)	103.43 ± 12.69 99.50 (97.50–115.00)	**0.002**
HDL [mg/dL]	49.33 ± 12.82 46.00 (41.00–54.00)	60.71 ± 19.09 60.50 (45.00–75.50)	**0.013**
TG [mg/dL]	135.18 ± 74.95 119.00 (92.00–163.00)	109.96 ± 26.19 110.50 (92.00–120.00)	0.271
Weight [kg]	77.77 ± 12.71 77.00 (69.00–85.00)	73.93 ± 10.02 75.00 (66.00–79.50)	0.245
BMI [kg/m^2^]	24.53 ± 3.30 24.00 (22.30–26.60)	23.03 ± 1.43 22.90 (22.05–24.25)	**0.059**

Abbreviations: BMI—body mass index, calculated as weight in kilograms divided by height in meters squared; FBG—fasting blood glucose concentration; HDL—high-density lipoprotein cholesterol; IQR—interquartile range, representing the range between the 25th and 75th percentiles of the data distribution; LDL—low-density lipoprotein cholesterol; n—number of participants included in the analysis; TC—total cholesterol concentration; TG—triglyceride concentration.; * *p* < 0.05 was considered statistically significant. Differences between groups were assessed using the Mann–Whitney U test, a non-parametric method used for comparisons of independent groups. Data are presented as median (interquartile range, IQR) unless otherwise indicated.

**Table 4:** Results of adipokines in the plasma of the study and the control group with statistical analysis.

Adipokines	Study Group (n = 53)	Control Group (n = 33)	*p* *
	Mean ± SD Median (IQR)		
Wnt5a [ng/mL]	2.91 ± 3.90 1.63 (1.23–2.07)	1.91 ± 0.60 1.72 (1.44–2.30)	0.421
ANGPTL2 [ng/mL]	5.29 ± 4.78 3.66 (2.91–5.42)	4.05 ± 2.35 2.96 (2.27–5.76)	**0.042**
Visfatin [ng/mL]	2.40 ± 0.91 2.40 (1.80–1.80)	4.09 ± 0.84 4.10 (2.20–5.60)	**<0.001**
LCN2 [ng/mL]	19.71 ± 8.69 16.69 (14.27–24.88)	20.02 ± 5.16 19.42 (16.09–23.06)	0.575
Resistin [ng/mL]	4.60 ± 2.69 3.90 (3.09–5.13)	4.18 ± 1.17 4.28 (3.40–4.90)	0.613
Chemerin [pg/mL]	91.91 ± 24.84 90.69 (76.76–106.45)	77.02 ± 19.62 85.52 (61.61–93.52)	0.104
SFRP5 [ng/mL]	20.07 ± 22.84 12.50 (11.00–16.00)	41.81 ± 55.25 15.50 (13–46)	**0.036**
Adiponectin [µg/mL]	7.65 ± 4.98 7.034 (4.24–9.00)	9.20 ± 3.13 9.07 (7.06–11.85)	**0.017**
Vaspin [pg/mL]	491.47 ± 339.96 443.18 (247.73–668.18)	346 ± 246.06 265.90 (172.72–463.63)	**0.042**
Omentin [pg/mL]	250 ± 242.03 135.47 (118.11–239.99)	236.55 ± 173.8 126.82 (114.12–272.55)	0.651
Apelin [ng/mL]	2.04 ± 1.96 1.27 (1.08–1.64)	1.80 ± 1.01 1.30 (1.15–2.14)	0.442

Abbreviation: Wnt5a—protein of Wnt Family Member 5A, ANGPTL2—angiopoietin related protein 2, LCN2—lipocalin 2, SFRP5—secreted frizzled related protein 5; * *p* < 0.05 was considered statistically significant. Differences between groups were assessed using the Mann–Whitney U test, a non-parametric method used for comparisons of independent groups. Data are presented as median (interquartile range, IQR) unless otherwise indicated.

**Table 5:** Results of demographic, biochemical and immunological indicators in subgroups of patients treated with two cART regimens.

Indicators	NRTI +INSTI (n = 30)	NRTI +PI (n = 23)	*p* *
	Mean ± SD Median (IQR)		
Age [years]	38.00 ± 7.14 37.00 (33.00–42.00)	41.39 ± 8.21 39.00 (34.00–45.00)	0.211
CD4+T lymphocytes [cells/µL]	692.09 ± 238.61 659.50 (549.00–842.00)	622.35 ± 267.25 615.50 (462.50–727.00)	0.222
CD8+T lymphocytes [cells/µL]	673.68 ± 204.12 686.50 (589.00–822.00)	745.60 ± 291.08 669.00 (514.00–974.00)	0.624
CD4+/CD8+ ratio	1.09 ± 0.46 0.98 (0.830–1.32)	0.95 ± 0.07 0.84 (0.58–1.31)	0.221
FBG [mg/dL]	93.42 ± 12.58 90.00 (87.00–104.00)	95.83 ± 18.06 92.00 (86.00–1.3.00)	0.988
TC [mg/dL]	186.76 ± 35.66 179.00 (172.00–197.00)	193.67 ± 40.32 198.50 (167.00–222.00)	0.414
LDL [mg/dL]	127.42 ± 42.71 113.00 (109.00–128.00)	128.39 ± 44.32 125.50 (81.00–156.00)	0.662
HDL [mg/dL]	49.80 ± 10.10 46.00 (44.00–55.00)	48.78 ± 15.32 47.50 (40.00–52.00)	0.489
TG [mg/dL]	120.90 ± 50.41 121.00 (78.00–151.00)	151.83 ± 94.32 116.50 (94.00–222.00)	0.489
Weight [kg]	78.03 ± 12.37 78.00 (70.00–85.00)	77.43 ± 13.32 76.00 (69.00–86.00)	0.672
BMI [kg/m^2^]	24.50 ± 3.25 23.75 (22.00–26.60)	24.57 ± 3.03 24.00 (22.30–27.00)	0.998

Abbreviations: BMI—body mass index, calculated as weight in kilograms divided by height in meters squared; FBG—fasting blood glucose concentration; HDL—high-density lipoprotein cholesterol; IQR—interquartile range, representing the range between the 25th and 75th percentiles of the data distribution; LDL—low-density lipoprotein cholesterol; n—number of participants included in the analysis; TC—total cholesterol concentration; TG—triglyceride concentration.; CD4^+^ T lymphocytes—cluster of differentiation 4-positive T helper lymphocytes, key immune cells involved in immune regulation and antiviral responses; CD8^+^ T lymphocytes—cluster of differentiation 8-positive cytotoxic T lymphocytes, responsible for the elimination of infected cells; CD4^+^/CD8^+^ ratio—ratio of CD4^+^ helper T lymphocyte count to CD8^+^ cytotoxic T lymphocyte count, used as an indicator of immune system balance; cells/μL—number of cells per microliter of blood; * *p* < 0.05 was considered statistically significant. Differences between groups were assessed using the Mann–Whitney U test, a non-parametric method used for comparisons of independent groups.

**Table 6:** Adipokines results in the study group depending on the type of cART.

Adipokins	NRTI + INSTI (n = 30)	NRTI +PI (n = 23)	*p* *
	Mean ± SD Me (IQR)		
Wnt5a [ng/mL]	3.78 ± 5.01 1.50 (1.29–2.55)	1.66 ± 0.32 1.26 (1.13–1.87)	0.080 INSTI vs. PI (*p* = 0.29) INSTI vs. C (*p* = 1.00) PI vs. C (*p* = 0.08)
ANGPTL2 [ng/mL]	6.32 ± 5.99 3.84 (3.06–6.34)	3.97 ± 1.23 3.25 (2.59–4.66)	0.089 INSTI vs. PI (*p* = 0.42) INSTI vs. C (*p* = 0.09) PI vs. C (*p* = 1.00)
Visfatin [ng/mL]	2.35 ± 1.02 2.20 (1.60–2.60)	2.47 ± 0.24 2.50 (1.90–2.90)	**<0.001** INSTI vs. PI (*p* = 1.00) **INSTI vs. C (*p* < 0.001)** **PI vs. C (*p* = 0.02)**
LCN2 [ng/mL]	17.17 ± 1.85 16.39 (13.96–19.12)	23.01 ± 11.56 20.33 (14.27–28.21)	0.067 INSTI vs. PI (*p* = 0.14) INSTI vs. C (*p* = 1.00) PI vs. C (*p* = 1.00)
Resistin [ng/mL]	4.20 ± 1.85 3.77 (2.76–5.12)	5.13 ± 3.12 4.37 (3.19–5.56)	0.574 INSTI vs. PI (*p* = 0.92) INSTI vs. C (*p* = 1.00) PI vs. C (*p* = 1.00)
Chemerin [pg/mL]	90.71 ± 21.49 90.69 (78.17–99.38)	93.46 ± 29.32 89.89 (67.67–108.07)	0.079 INSTI vs. PI (*p* = 1.00) INSTI vs. C (*p* = 0.11) PI vs. C (*p* = 0.29)
SFRP5 [ng/mL]	26.88 ± 35.66 12.50 (10.50–19.50)	15.52 ± 8.21 13.00 (11.00–16.00)	**0.028** INSTI vs. PI (*p* = 1.00) INSTI vs. C (*p* = 0.05) PI vs. C (*p* = 0.09)
Adiponectin [µg/mL]	6.29 ± 4.25 6.25 (3.00–8.33)	9.42 ± 5.12 8.51 (4.76–11.88)	**0.003** INSTI vs. PI (*p* = 0.04) **INSTI vs. C (*p* < 0.001)** PI vs. C (*p* = 1.00)
Vaspin [pg/mL]	492.87 ± 353.66 444.31 (265.90–606.81)	489.62 ± 329.03 325.00 (247.72–704.55)	0.110 INSTI vs. PI (*p* = 1.00) INSTI vs. C (*p* = 0.23) PI vs. C (*p* = 0.22)
Omentin [pg/mL]	276.72 ± 271.01 143.18 (123.09–297.10)	217.12 ± 198.00 127.99 (107.81–230.80)	0.217 INSTI vs. PI (*p* = 0.25) INSTI vs. C (*p* = 0.0.91) PI vs. C (*p* = 1.00)
Apelin [ng/mL]	2.49 ± 2.46 1.30 (1.02–2.56)	1.45 ± 0.21 1.24 (1.08–1.43)	0.363 INSTI vs. PI (*p* = 1.00) INSTI vs. C (*p* = 1.00) PI vs. C (*p* = 0.49)

Abbreviation: Wnt5a—protein of Wnt Family Member 5A, ANGPTL2—angiopoietin related protein 2, LCN2—lipocalin 2, SFRP5—secreted frizzled related protein 5; NRTIs—nucleoside/nucleotide reverse transcriptase inhibitors, a class of antiretroviral drugs that inhibit HIV reverse transcriptase and prevent viral DNA synthesis; INSTIs—integrase strand transfer inhibitors, antiretroviral agents that block integration of viral DNA into the host genome; PIs—protease inhibitors, antiretroviral drugs that inhibit HIV protease activity and prevent the formation of mature infectious viral particles; * *p* < 0.05 was considered statistically significant. Differences among multiple independent groups were assessed using the Kruskal–Wallis test followed by Dunn’s post hoc test for pairwise comparisons; C—Concentration of adipokines in the control group are posted in Table 3.

**Table 7:** Correlations coefficients between adipokines with adipokines and biochemical immunological indices in the study group.

**Adipokins vs. Adipokins**	***R***	***p***	
SFRP5 vs. apelin	0.764	<0.001	high correlation
ANGPTL2 vs. apelin	0.753	<0.001	
Wnt5a vs. apelin	0.745	<0.001	
ANGPTL2 vs. SFRP5	0.714	<0.001	
Wnt5a vs. ANGPTL2	0.563	<0.001	
Wnt5a vs. SRRP5	0.544	<0.001	
Wnt5a vs. omentin	0.476	<0.001	average correlation
SFRP5 vs. omentin	0.415	0.002	
Omentin vs. apelin	0.399	0.003	
ANGPTL2 vs. omentin	0.386	0.004	
**Adipokins vs. other indicators**	***R***	***p***	
Wnt5a vs. age	−0.403	0.003	average correlation
SFRP5 vs. age	−0.403	0.003	
LCN2 vs. CT	0.374	0.019	
Vaspin vs. BMI	0.320	0.020	
Vaspin vs. weight	0.304	0.027	

Abbreviation: Wnt5a—protein of Wnt Family Member 5A, ANGPTL2—angiopoietin related protein 2, LCN2— lipocalin 2, SFRP5—secreted frizzled related protein 5; *R—Spearman’s* rank correlation coefficient, indicating the strength and direction of the correlation between analyzed variables (positive values indicate a direct association, whereas negative values indicate an inverse association); *p*–value indicating the statistical significance of the observed correlation. Correlations with *p* < 0.05 were considered statistically significant.

**Table 8:** Correlations between adipokine concentrations and biochemical and immunological parameters, age, body weight, and BMI in patients receiving two different cART regimens.

ISTI + NRTI			PI + NRTI		
**Adipokines vs. Adipokines**	***R***	***p***	**Adipokines vs.** **Adipokines**	***R***	***p***
Wnt5a vs. apelin	0.899	<0.001	ANGPTL2 vs. apelin	0.632	0.001
ANGPTL2 vs. SFRP5	0.889	<0.001	Wnt5a vs. apelin	0.626	0.001
SFRP5 vs. apelin	0.855	<0.001	SFRP5 vs. apelin	0.586	0.003
Wnt5a vs. SFRP5	0.853	<0.001	Wnt5a vs. SFRP5	0.533	0.009
ANGPTL2 vs. apelin	0.822	<0.001	Resistin vs. adiponectin	0.518	0.011
Wnt5a vs. ANGPTL2	0.795	<0.001	ANGPTL2 vs. SFRP5	0.151	0.012
SFRP5 vs. omentin	0.639	0.002	Vaspin vs. omentin	−0.479	0.021
Omentin vs. apelin	0.621	<0.001	Wnt5a vs. chemerin	−0.448	0.032
Wnt5a vs. omentin	0.541	0.002	Resestin vs. chemerin	0.446	0.024
ANGPTL2 vs. omentin	0.506	0.004	Resistin vs. vaspin	−0.425	0.043
Wnt5a vs. LCN2	0.444	0.014	Adiponektin vs. vaspin	−0.422	0.045
Visfatin vs. chemerin	0.409	0.024			
**Adipokines vs. other indicators**	***R***	***p***	**Adipokines vs. other indicators**	***R***	***p***
LCN2 vs. CD4+	0.619	0.002	LCN2 vs. CT	0.587	0.010
Wnt5a vs. age	0.466	0.009	Resistin vs. LDL	−0.538	0.023
SFRP5 vs. CD8+	−0.465	0.029	Wnt5a vs. CD8+	0.502	0.020
Visfatin vs. LDL	0.463	0.034	ANGPTL2 vs. CD4+	−0.459	0.042
SFRP5 vs. age	−0.447	0.013	Wnt5a vs. index CD4+/CD8+	−0.444	0.049
Visfatin vs. CT	0.438	0.047	LCN2 vs. BMI	0.443	0.034
Visfatin vs. BMI	0.393	0.032	Visfatin vs. age	−0.441	0.035
Wnt5a vs. weight	−0.374	0.042			
Vaspin vs. weight	0.364	0.048			
Apelin vs. age	−0.364	0.048			
Apelin vs. age	−0.364	0.048			

Abbreviation: Wnt5a—protein of Wnt Family Member 5A, ANGPTL2—angiopoietin related protein 2, LCN2—lipocalin 2, SFRP5—secreted frizzled related protein 5; *R*—Spearman’s rank correlation coefficient, indicating the strength and direction of the correlation between analyzed variables (positive values indicate a direct association, whereas negative values indicate an inverse association); *p*–value indicating the statistical significance of the observed correlation. Correlations with *p* < 0.05 were considered statistically significant.

## Data Availability

All data generated or analysed during this study are included in the manuscript. Further inquiries should be directed to the corresponding author.

## Abbreviations

ANGPTL2	angiopoietin-like protein 2
BMI	body weight and body mass index
cART	combination antiretroviral therapy
FBG	fasting blood glucose
HDL	high-density lipoprotein
HIV	human immunodeficiency virus
INSTI	integrase strand transfer inhibitors
LCN2	lipocalin 2
LDL	low-density lipoprotein
NRTI	nucleoside reverse transcriptase inhibitor
PI	protease inhibitors
SFRP5	secreted frizzled-related protein 5
TC	total cholesterol
TG	triglycerides
Wnt5a	Wnt family member 5A
